# Reproductive Costs Increase With Longer Extreme Heat Events in Collembola

**DOI:** 10.1002/ece3.71775

**Published:** 2025-07-09

**Authors:** Anouk Gremion, Madhav P. Thakur, Gerard Martínez‐De León

**Affiliations:** ^1^ Institute of Ecology and Evolution University of Bern Bern Switzerland

**Keywords:** egg viability, fecundity, *Folsomia candida*, *Proisotoma minuta*, recovery, warming

## Abstract

Temperature regimes are changing at an unprecedented rate, leading to more intense, frequent, and prolonged extreme heat events. These conditions can undermine the performance of organisms during and after extreme events. Yet, our understanding of how different durations of extreme heat events impact the reproductive traits of soil invertebrates is limited. Here, we experimentally tested how exposure to extreme heat (30°C–26°C, day–night) affects the reproductive recovery of two Collembola species, *Folsomia candida* (parthenogenetic) and *Proisotoma minuta* (sexually reproducing), originally reared for several generations at two different source temperatures (15°C and 20°C). We exposed these collembolans to extreme heat events of varying durations (0, 2, 4 or 8 days), and allowed them to recover for 2 days at their respective source temperatures. We then examined how reproductive traits (offspring production and egg sizes) were affected by previous exposure to extreme heat of varying duration. We predicted that prolonged heat events would have greater impacts on the reproductive traits of both Collembola species, particularly for collembolans raised in colder environments (due to lower acclimation to warming) and in traits related to offspring viability (due to their higher thermal sensitivity). Our results show that offspring production—number of eggs and hatchlings—declined with longer exposure to extreme heat in both species. Collembolans raised in the warmer environment had a steeper reduction in the number of eggs with longer exposure to heat, whereas those reared in the colder environment showed more marked reductions in the number of hatchlings (with declines of up to 99% in cold and 49% in warm rearing environments in *F. candida* ), indicating heat‐induced reductions in egg viability. Overall, our results demonstrate that longer exposure to extreme heat reduces the reproductive success of collembolans, with larger fitness consequences for those from colder environments.

## Graphical abstract

We provide experimental evidence for how acclimation temperatures can modulate the impacts of prolonged extreme heat events on reproductive traits, with stronger effects for individuals raised in colder environments. These findings contribute to our mechanistic understanding of extreme heat impacts on reproduction, with direct implications for population‐level responses.

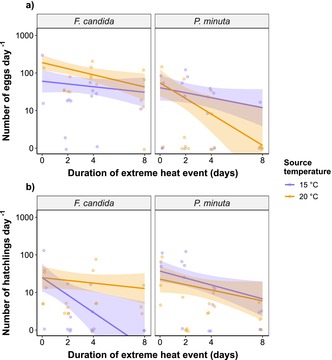

## 1 Introduction

Climate change is associated with an increased frequency and severity of climate extremes, such as extreme heat events (Buckley and Huey 2016; Fischer et al. 2021; IPCC 2023). Understanding the ecological impacts of extreme heat events is crucial to determining how climate change might alter biodiversity dynamics in the long run (Harris et al. 2018; Harvey et al. 2022; Thakur et al. 2022). Temperature extremes can disrupt the physiological condition and induce mortality in some organisms (González‐Tokman et al. 2020; Ma et al. 2020; Williams et al. 2016), which implies that more frequent extreme events can drive population declines and range contractions owing to heat stress (Jørgensen et al. 2022). Ectothermic animals are especially vulnerable to extreme heat events since their body temperatures closely track environmental temperatures. In this regard, their responses to increasing intensity, frequency, and duration of extreme heat events will have consequences for their life‐history traits and ultimately their fitness.

Extreme heat events can induce shifts in life‐history traits as a result of physiological heat damage and allocation trade‐offs (Martínez‐De León and Thakur 2024). Among life‐history traits, reproductive traits are considered to be particularly vulnerable to elevated temperatures (Walsh et al. 2019), as their thermal limits are often much lower than those of other traits, such as survival (Bozinovic et al. 2020; Martínez‐De León, Marty, et al. 2024; van Heerwaarden and Sgrò 2021). Moreover, heat exposure can impair a number of behavioral (Isotalo et al. 2022; Pilakouta et al. 2023; Zizzari and Ellers 2011) and physiological processes related to ectotherms' reproduction (Aprison and Ruvinsky 2014; Breedveld et al. 2023; Canal Domenech and Fricke 2022; Sales et al. 2021), collectively leading to declines in their reproductive success with subsequent reductions in population sizes (Martínez‐De León, Marty, Holmstrup, et al. 2024). Once temperatures return to normal at the end of an extreme heat event, physiological recovery of reproductive traits begins (Aprison and Ruvinsky 2014; Canal Domenech and Fricke 2022; Jørgensen et al. 2006; Sales et al. 2021), allowing vital rates to return to levels prior to the extreme heat event (Ma et al. 2018). Therefore, the consequences of heat extremes for population dynamics are not only influenced by the effects manifested during the extreme event, but also by the speed of recovery of reproductive traits once stressful conditions have ended (Martínez‐De León and Thakur 2024). Whether reproductive traits fully recover could depend on the accumulation of heat stress (Ørsted et al. 2022), which is likely related to the duration of an ectotherm's exposure to an extreme heat event (Aprison and Ruvinsky 2014; Isotalo et al. 2022; Jørgensen et al. 2006; Ma et al. 2018; Xie et al. 2023). Longer exposure to extreme heat could indeed lead to irreversible heat injuries, particularly during early development (Canal Domenech and Fricke 2022), while shorter exposure may only have transient impacts that do not affect the viability of future offspring (Jørgensen et al. 2006; Walsh et al. 2021). Therefore, longer‐lasting extreme heat events could have persistent effects on reproductive traits even after the event has ended. Given the importance of reproductive success for population persistence, gaining knowledge on the ability of species to recover after extreme heat events is crucial for our mechanistic understanding of the impacts of extreme heat on biodiversity.

The impacts of extreme heat on reproductive traits could further depend on the thermal conditions to which organisms are acclimated. For instance, organisms raised in warmer environments can downregulate their metabolic and physiological rates (Seebacher et al. 2015; Wootton et al. 2022), have smaller adult body sizes (Ohlberger 2013; Sentis et al. 2024), and allocate more resources to reproduction than in colder environments (Fryxell et al. 2020; Wootton et al. 2022), which could confer them higher fitness under heat exposure (Vasudeva et al. 2019). However, being acclimated at elevated temperatures might not always help organisms to withstand heat extremes better, as previously shown for several performance traits (Tüzün and Stoks 2018) including those related to reproductive success, such as egg viability (Liefting et al. 2010). The environmental temperatures experienced by organisms after extreme heat events could also play a role in their recovery. For instance, physiological recovery has been suggested to be temperature‐dependent (Ørsted et al. 2022), which means that warmer environments (within the permissive thermal range) could provide faster recovery after heat extremes (Bowler and Kashmeery 1979), or alternatively, constrain recovery if physiological damage continues to accumulate in warm environments (Kefford et al. 2022; Ørsted et al. 2022). In any case, the rate of recovery may again depend on the thermal conditions to which organisms are acclimated.

In this study, we investigated how the recovery of reproductive traits (~2 days after extreme heat) is affected by extreme heat events of varying duration (0–8 days). We explored this question using two species of soil‐living Collembola ( *Folsomia candida* and *Proisotoma minuta* , family Isotomidae) reared at different thermal environments (source temperatures of 15°C or 20°C). Given the widespread occurrence of Collembola across diverse climatic conditions worldwide (Potapov et al. 2023), investigating their thermal responses to extreme heat events could provide valuable insights into how reproductive traits of soil‐living ectotherms are affected by varying exposure to heat extremes. The two species used in our experiments differ in a number of traits, such as in their mode of reproduction: *F. candida* is parthenogenetic, while *P. minuta* reproduces sexually. Sexual reproduction may particularly be heat‐sensitive due to the involvement of more complex physiological and behavioral processes (e.g., sperm production and fertilization; Walsh et al. 2019), which may also take longer to recover. However, parthenogenesis in *F. candida* is induced by the obligate endosymbiotic bacterium *Wolbachia*, which is itself sensitive to high temperatures and requires some time to reestablish after being exposed to extreme heat (Timmermans and Ellers 2009). We hypothesize (1) that longer durations of extreme heat result in reduced recovery of offspring production (e.g., number of eggs and hatchlings), particularly in organisms acclimated to colder conditions. We expect (2) similar responses in the two Collembola species despite their distinct reproductive modes, especially in terms of the production of viable offspring. This is because, in both species, hatching success (i.e., viable offspring) is possibly more heat‐sensitive than egg production, due to the necessary role of *Wolbachia* for egg hatching in *F. candida* and the need for successful fertilization in *P. minuta* (Timmermans and Ellers 2009; Zizzari and Ellers 2011). Finally, we predict that (3) egg sizes, a proxy of offspring provisioning, will shrink after longer exposure to extreme heat, as a result of heat damage during oogenesis or due to reduced maternal investments.

## 2 Methods

### 2.1 Study Species

Two Collembola species belonging to the family Isotomidae, *F. candida* and *P. minuta*, were used in this experiment. These collembolans are litter‐ and soil‐living species, occurring in several kinds of temperate habitats, such as forests and agricultural soils (Fountain and Hopkin 2005; Gisin 1943). The populations of *F. candida* consist of parthenogenetic females (Czarnetzki and Tebbe 2004; Fountain and Hopkin 2005), while *P. minuta* reproduces sexually. The cultures of both species were kept under constant dark conditions in plastic containers with a moist substrate of plaster of Paris and charcoal, with dry yeast as a food source. The two species have been maintained in separate batch cultures at 15°C and 20°C (source temperatures) for 2 years before the start of the experiment (representing 20–30 generations; Martínez‐De León, Fahrni, and Thakur 2024). Additional details of the origin of the cultures are provided in Marty et al. (2022).

### 2.2 Experimental Design

We exposed adult collembolans originating from the two source temperatures (15°C or 20°C) to extreme heat events of varying duration: 0, 2, 4, and 8 days. Each treatment was replicated five times, resulting in 80 experimental units (2 species × 2 source temperatures × 4 durations × 5 replicates). The extreme heat treatment consisted of diel cycles of 30°C during the day (16 h) and 26°C during the night (8 h) in dark incubators (SANYO MIR‐253, Japan) to mimic daily temperature fluctuations. These temperatures are known to impact reproductive traits in Collembola, such as fecundity (Mallard et al. 2020; Martínez‐De León, Marty, Holmstrup, et al. 2024) and egg viability (Fountain and Hopkin 2005; Martínez‐De León, Fahrni, and Thakur 2024). In addition, these conditions are representative of current and future heat wave scenarios in several temperate soils (IPCC 2023; Lembrechts et al. 2022) where the species can potentially occur (Gisin 1943). The experimental units consisted of glass plates (∅ 5 cm) with a moist substrate of plaster of Paris and activated charcoal (9:1 mixture). We added 20 adult individuals to each plate, randomly sampled from the cultures, and fed them with dry yeast provided *ad libitum*. Sex determination in living individuals of the sexually reproducing *P. minuta* was not feasible in our study, as it would have required the examination of the genital aperture on microscopic slides (cf. Chahartaghi et al. (2006)). Therefore, it was not possible to control the sex ratios of the living *P. minuta* that were inoculated in the experimental units.

**FIGURE 1:**
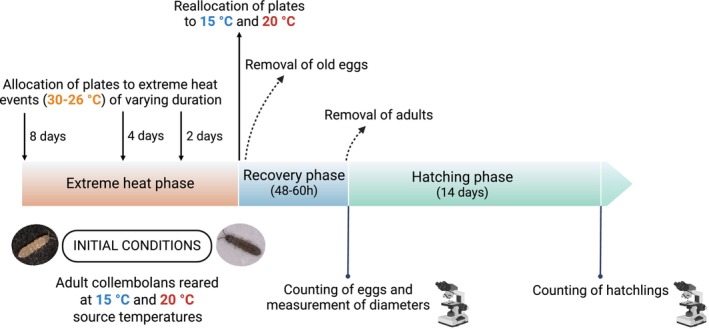
Timeline of the experiment. Several measurements were taken at each experimental phase: Extreme heat phase (total number of eggs during exposure to heat), recovery phase (total number of eggs, clutch size, number of clutches, egg diameter) and hatching phase (number of hatchlings). The 0‐day treatment samples (i.e., controls without exposure to extreme heat) were kept in the incubators set at the respective source temperatures (15°C or 20°C) during the whole experiment. The pictures show the two Collembola species used in the experiment: *Folsomia candida* (left picture) and *Proisotoma minuta* (right picture). Created with biorender.com.

The experiment consisted of three phases: extreme heat phase, recovery phase, and hatching phase (Figure 1). In the extreme heat phase, the experimental units were sequentially transferred to the incubator set at extreme heat conditions, starting with the samples allocated to the longest duration of the heat event (8 days) until the shortest one (2 days). All experimental units ended their exposure to extreme heat simultaneously and were brought together back to the incubators set at their respective source temperatures (15°C or 20°C) (Figure 1). The samples allocated to the 0‐day treatment (i.e., without exposure to extreme heat) were kept in the incubators set at the respective source temperatures during the entire duration of the experiment. Next, we counted and removed the eggs previously produced during the extreme heat event phase (Figure S2) and allowed the adults to oviposit at their respective source temperatures during 48–60 h (recovery phase). Given that collembolans alternate reproductive with non‐reproductive instars in intervals of several days (Fountain and Hopkin 2005), we expected that only one reproductive event per female (i.e., laying of one clutch) could take place during the recovery phase. Thus, the recovery phase served as a standardized sampling interval across treatments, despite differences in the duration of extreme heat exposure. After this period, we counted and removed all the adults and estimated the number of clutches (i.e., discrete clusters of at least 10 eggs), the number of eggs per clutch (i.e., clutch size; Moretti et al. 2017) and egg sizes (diameters of the eggs measured at 50X magnification; VHX‐970f, KEYENCE Corp., Japan), leaving them untouched to avoid undesired impacts of handling on egg viability. This implies that the number of eggs was likely underestimated owing to imperfect detection (e.g., eggs buried in holes in the substrate) and that only eggs with visible diameters were measured (Figure S3). Finally, in the hatching phase, hatchlings were counted weekly until no further eggs hatched (i.e., 2 weeks after the first observed hatching event), which marked the end of the experiment (Figure 1).

### 2.3 Replication Statement

**Table:** 

Scale of inference	Scale at which the factor of interest is applied	Number of replicates at the appropriate scale
Experimental unit	Plate	5

### 2.4 Data Analysis

We analyzed how reproductive traits measured at the recovery and hatching phases (recovery phase: total number of eggs, clutch size, number of clutches, egg diameter; hatching phase: number of hatchlings) were influenced by extreme heat duration, species, source temperature, and their interactions (R package glmmTMB; Brooks et al. 2017). All response variables except for egg diameter were analyzed using generalized linear models (GLM) with a negative binomial distribution. We restricted the analysis of clutches to groupings with at least 10 eggs, and in the case of clutch sizes, we accounted for multiple measurements in the same experimental unit by treating plate ID as a random effect. Egg diameter was analyzed with linear mixed‐effect models, additionally adding clutch ID as a random effect. We then used the function *emtrends* (R package emmeans; Lenth 2024) as a post hoc analysis to test whether the slopes from the linear models—describing the relationship between the response variables and extreme heat duration—differed from zero, and how they varied between source temperatures and species. As a proxy for the impacts of extreme heat on egg viability, we compared how the slopes of egg and hatchling numbers varied with the duration of extreme heat. We additionally tested whether the proportion of surviving adults was affected by the extreme heat events using a binomial GLM and whether including the number of surviving adults as an additive term influenced offspring production in the GLMs. Eggs produced during the extreme heat phase were counted but not analyzed, given that the duration of the extreme heat event can be confounded with the time interval in which females were allowed to lay eggs (Figure S1). We visually assessed and verified linearity assumptions of all models with the DHARMa package (Hartig 2022). All statistical analyses were conducted in R (R Core Team 2024).

## 3 Results

**FIGURE 2:**
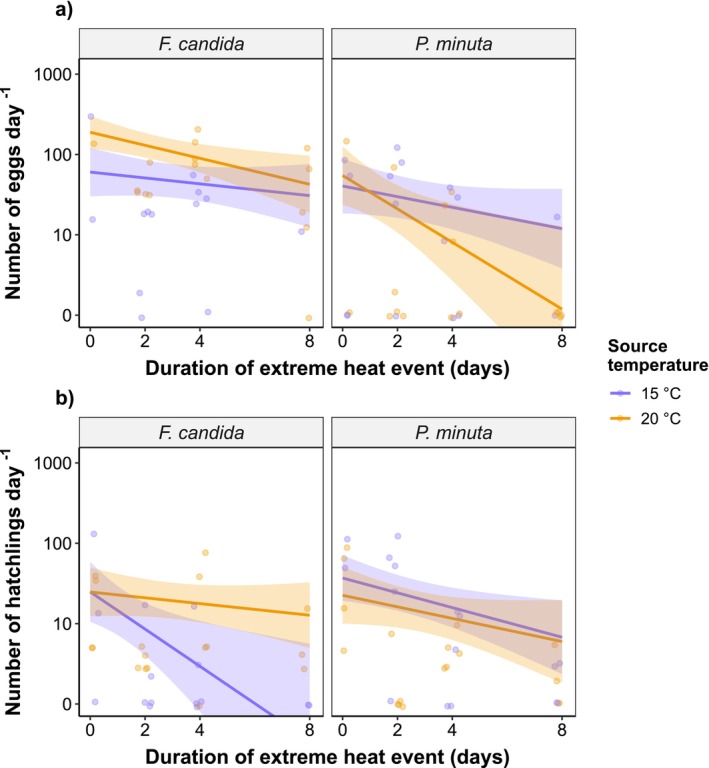
Predicted number of eggs (a) and hatchlings (b) with 95% confidence intervals (CI) in response to extreme heat events of increasing duration. Colors designate the different source temperatures used for the rearing of Collembola adults: 15°C (blue) or 20°C (orange). Faded points represent raw data. Note the log‐10 scaling in the *y*‐axis. Full model outputs are provided in Tables S1–S4.

**FIGURE 3:**
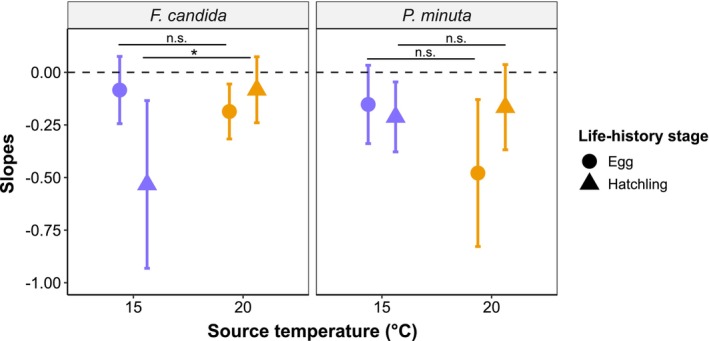
Slopes with 95% confidence intervals (CI) of the regression lines shown in Figure 2, describing the responses of egg (circles) and hatching numbers (triangles) to increasing durations of extreme heat events. Colors designate the different source temperatures used for the rearing of Collembola adults: 15°C (blue) or 20°C (orange). Please note that life‐history stages were studied at the recovery phase (eggs) and the hatchling phase (Figure 1). Confidence intervals that do not overlap with zero (broken line) imply significant effects of extreme heat duration. The star sign (*) indicates significant differences between the slopes of different source temperatures at *p <* 0.05. Full model outputs, including the intercepts, are provided in Tables S1–S4.

**FIGURE 4:**
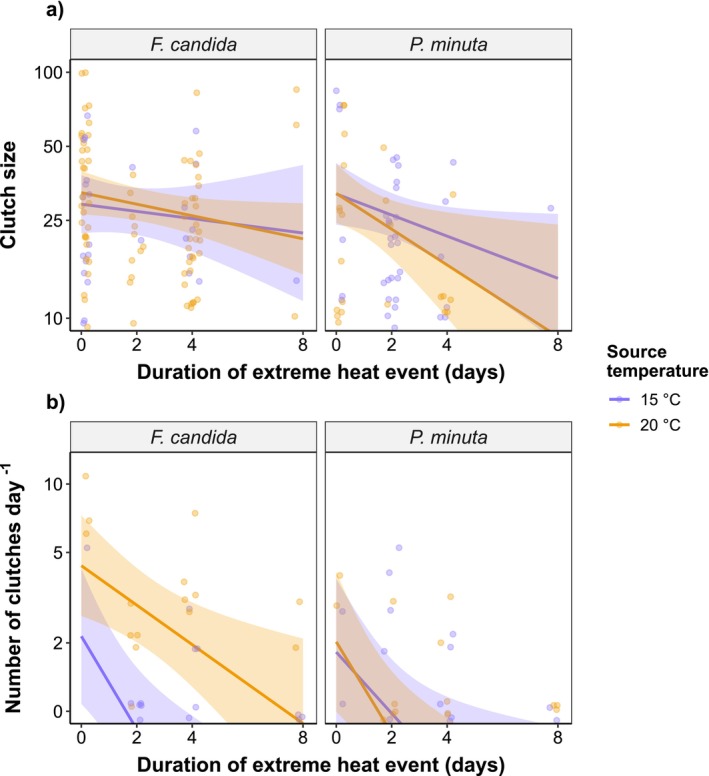
Predicted clutch sizes (a) and number of clutches (b) with 95% confidence intervals (CI) in response to extreme heat events of increasing duration. Colors designate different source temperatures at which the adults used in the experiments were raised: 15°C (blue) or 20°C (orange). Faded points represent raw data. Note the log‐10 scaling in the y‐axis. Full model outputs are provided in Tables S5–S8.

After a recovery period of ~2 days, collembolans exposed to longer durations of extreme heat produced fewer eggs, especially those raised at the higher source temperature of 20°C (95% CI of the GLM slope, *F. candida* : [−0.450, −0.067]; *P. minuta* : [−0.562, −0.112]; Figure 2a; Figure 3; Tables S1 and S2). In this case, egg production dropped by −77.5% in *F. candida* and by −97.8% in *P. minuta* when increasing the duration of extreme heat from 0 to 8 days. This decline in egg production was mostly driven by a reduction in the frequency of egg laying events (i.e., number of clutches). Specifically, while clutch sizes were only weakly affected (e.g., −35.1% from 0 to 8 days of exposure in *F. candida* from 20°C source; Figure 4a; Tables S5 and S6), longer heat events substantially hindered the number of clutches produced (e.g., −79.8% from 0 to 8 days of exposure in *F. candida* from 20°C source; Figure 4b; Tables S7 and S8). Likewise, the number of hatchlings declined with extreme heat duration, but mainly at the source temperature of 15°C (95% CI of the GLM slope, *F. candida* : [−0.931, −0.134]; *P. minuta* : [−0.378, −0.046]; Figure 2b; Figure 3; Tables S3 and S4). In this context, the number of hatchlings declined by −98.6% in *F. candida* —practically causing a complete reproductive failure—and by −74.3% in *P. minuta* when increasing the duration of extreme heat from 0 to 8 days. Overall, both *F. candida* and *P. minuta* showed similar patterns in their responses to extreme heat duration (Figure 3). However, the effect of extreme heat duration (i.e., slopes of the GLMs) differed significantly between source temperatures in *F. candida* at the hatchling stage, with the 15°C source temperature inducing more negative responses (difference in GLM slopes between source temperatures ±SE: 0.450 ± 0.218; *p =* 0.039; Figure 3). All adult *F. candida* survived until the end of the short‐term recovery period, whereas mortality was higher in *P. minuta* , but unrelated to the duration of the extreme heat events or to the source temperature (Figure S1). We did not find any significant influence of the number of surviving adults on offspring production at the end of the recovery phase (estimate GLM ± SE; eggs: 0.229 ± 0.171, *p* = 0.178; hatchlings: 0.184 ± 0.119, *p* = 0.122). Similarly, we did not detect any effects of extreme heat duration on egg diameter (Figure S3).

## 4 Discussion

Our findings indicate that longer extreme heat events hinder the reproductive success of two Collembola species after a 2‐day recovery period at favorable temperatures. This 2‐day recovery represents an immediate egg laying event following the heat extremes. Importantly, exposure to the extreme heat events had major impacts on offspring production while having negligible effects on survival, underscoring the significance of reduced reproductive outputs as drivers of population dynamics after heat extremes. The effects of extreme heat on reproductive traits were modulated by the source temperatures experienced by adult collembolans through their development: the number of hatchlings declined more strongly with heat duration when the adult collembolans came from the colder environment (15°C), whereas the number of eggs was more affected by heat duration when the adults originated from the warmer environment (20°C). These findings suggest that reproductive traits may be differentially affected depending on the thermal environments previously experienced by organisms, with overall greater fitness consequences for organisms from colder environments.

### 4.1 Short‐Term Recovery of Reproductive Traits Is Hindered With Longer Exposure to Extreme Heat

The two species used in our experiments, the Collembola *F. candida* and *P. minuta* , showed similar declines in reproductive success as the duration of extreme heat events increased, suggesting that these responses could be widespread in other soil‐living ectotherms. These results confirm our initial hypothesis and match with previous findings on life‐history trade‐offs under heat exposure, namely that survival after heat stress can be sustained at the cost of reducing reproductive outputs (e.g., Krebs and Loeschcke 1994; Martínez‐De León, Marty, Holmstrup, et al. 2024). In our study, egg production was severely disrupted after ~2 days of recovery at favorable temperatures, especially in individuals coming from the warmer environment (20°C), indicating incomplete repair of heat damage on gametogenesis or transient sterility in some individuals. Since the number of clutches declined more strongly than clutch sizes (Figure 4; Tables S3 and S4), the observed decline in egg production was possibly driven by some individuals that were unable to reproduce during the recovery period. These results are in line with previous studies on male reproductive traits of *Drosophila* flies and *Tribolium* flour beetles, which point out greater impacts on reproductive success and more persistent fertility loss with longer exposure to extreme heat (Jørgensen et al. 2006; Sales et al. 2021). Similarly, it has been shown that the recovery of egg production after heat stress in the nematode *Caenorhabditis elegans* was hindered as a result of heat damage on the reproductive system, exacerbated by longer durations of extreme heat (Aprison and Ruvinsky 2014). In our study, the reproductive output of those individuals that managed to lay clutches was also reduced to some extent, consistent with past findings with the Collembola *Orchesella cincta* , showing that females produce smaller clutches after being exposed to acute heat stress (Zizzari and Ellers 2014).

Besides heat damage, it is also possible that reduced reproductive outputs are mediated by allocation trade‐offs, for instance as a result of enhanced expression of heat shock proteins (Krebs and Loeschcke 1994; Martínez‐De León and Thakur 2024). These protective mechanisms can confer improved survival by preserving protein structure and function (Sørensen et al. 2003). However, expressing heat shock proteins is energetically costly, which implies that fewer resources are available for non‐vital functions, such as growth or reproduction, until the synthesis of heat shock proteins is downregulated (Kingsolver and Woods 2016; Martínez‐De León and Thakur 2024).

### 4.2 Source Temperatures Modulate the Impacts of Extreme Heat Duration on Different Traits

We have shown that longer extreme heat events have stronger impacts on egg production in adults raised in the warmer thermal environment (20°C), while the number of hatchlings was more affected when adults originated from the colder environment (15°C) (Figure 4; Tables S1 and S2). These findings suggest that egg production and egg viability are affected in different ways depending on the thermal environment where the progenitors have developed. Although it is true that developing at warmer temperatures does not provide consistent benefits when confronted with heat extremes, as shown for egg production, the ultimate measure for reproductive success (i.e., production of viable offspring) was less affected in individuals coming from warmer source environments. We believe that our findings are not driven by hardening mechanisms that enhance performance under future heat stress, as the source temperatures fall within the permissible temperature range for both species (Mallard et al. 2020; Martínez‐De León, Fahrni, and Thakur 2024; Wehrli et al. 2024), and are therefore unlikely to trigger heat stress responses (Sørensen et al. 2003). Rather, it is possible that warmer environments for development may favor the production of viable offspring after heat extremes via other morphological or physiological adjustments, such as by enhancing egg provisioning (Pettersen et al. 2019) or by shifting the lipid membrane composition (van Dooremalen et al. 2011). Yet, egg size, a proxy of offspring quality (Liefting et al. 2010), did not differ between source temperatures or with the duration of extreme heat events (Figure S3) (Martínez‐De León, Fahrni, and Thakur 2024), indicating that other physiological mechanisms enhancing egg viability at elevated temperatures should be involved. Alternatively, it is possible that the thermal environment experienced during recovery affected the rates of physiological repair with concomitant effects on egg viability, implying that warmer environments provided faster repair of heat damage (Bowler and Kashmeery 1979; Ørsted et al. 2022). On the other hand, the causes of higher egg production during post‐heat recovery in adults developed in cooler environments are less clear. We speculate that cool‐acclimated adults sustained their reproductive activities despite exposure to heat stress (Gouvêa et al. 2015), which may come at the cost of subsequent failure in the production of viable gametes (Aprison and Ruvinsky 2014).

### 4.3 Longer Extreme Heat Events Had Similar Impacts on Collembola Species With Contrasting Reproductive Strategies

We confronted two Collembola species with different modes of reproduction to extreme heat events, to evaluate how heat exposure may have distinct effects on their reproductive traits. Our initial hypothesis was that reproductive traits would be similarly affected by extreme heat in the sexually reproducing ( *P. minuta* ) and in the parthenogenetic species ( *F. candida* ). This expectation was confirmed, as both species showed comparable thermal responses for the production of eggs and hatchlings. Yet, the number of hatchlings was particularly affected in *F. candida* from cooler source temperatures (Figure 3), resulting in almost complete reproductive failure (Figure 2b), which suggests that the negative impacts of heat on the occurrence of its obligate endosymbiont *Wolbachia* may not allow the production of viable eggs (Timmermans and Ellers 2009). Given that the prevalence of *Wolbachia* may take some time to recover (Timmermans and Ellers 2009), the viability of *F. candida* eggs can remain low even after heat events have ended. In *P. minuta* , egg production was comparably more impacted, especially in adults from warmer environments (Figure 3). This may suggest the occurrence of heat‐induced failure during gametogenesis or fertilization (Gouvêa et al. 2015; Zizzari and Ellers 2011), which could be supported by the lower thermal limits of male fertility in many sexually reproducing species (Sales et al. 2021; van Heerwaarden and Sgrò 2021). The sensitivity of reproduction in both Collembola species, even after the recovery period, could help to explain their responses to heat events at the population level, specifically the marked decline in population growth rates reported in *F. candida* after exposure to extreme heat (Martínez‐De León, Marty, and Thakur 2024). Even if our findings suggest similar thermal responses of the two studied species with contrasting reproductive modes (i.e., parthenogenesis and sexual reproduction), only extensive comparative studies will allow us to draw more robust conclusions about the thermal sensitivity of distinct reproductive strategies.

## 5 Conclusions

We demonstrated that longer exposure to extreme heat events has persistent consequences for the reproductive success of two soil‐living Collembola species with distinct modes of reproduction. These findings confirm that extreme heat events have deleterious effects on reproduction that can prevail even after temperatures return to normal, causing ecological debts at the population level (Martínez‐De León and Thakur 2024), at least in the short term. Periodic heat events can indeed amplify such reproductive debts, particularly when recovery periods become shorter. Our results contribute to a better understanding of the impacts of heat extremes on reproductive success and highlight the need for future studies on the long‐term recovery of reproductive traits (Canal Domenech and Fricke 2022; Jørgensen et al. 2006; Sales et al. 2021; Xie et al. 2023), as well as how recovery rates vary in different thermal environments (Ørsted et al. 2022).

## Author Contributions

**Anouk Gremion:** investigation (lead), writing – original draft (equal). **Madhav P. Thakur:** conceptualization (equal), funding acquisition (lead), methodology (equal), project administration (equal), resources (lead), supervision (equal), writing – review and editing (equal). **Gerard Martínez‐De León:** conceptualization (equal), data curation (lead), formal analysis (lead), investigation (supporting), methodology (equal), project administration (equal), supervision (equal), validation (lead), visualization (lead), writing – original draft (equal), writing – review and editing (equal).

## Conflicts of Interest

The authors declare no conflicts to declare.

## Supporting information


Appendix S1.

## Data Availability

The complete dataset and R script used in this study are available in the Figshare repository: https://doi.org/10.6084/m9.figshare.26978110.v1.
