# Supplementary material for: Reproductive Costs Increase With Longer Extreme Heat Events in Collembola
Source: Ecol Evol. 2025 Jul 9;15(7):e71775. doi: 10.1002/ece3.71775 (PMC12240591; doi:10.1002/ece3.71775)
Supplement: Supplementary file 1 — Appendix S1. [file ECE3-15-e71775-s001.docx]

**Supporting Information - Reproductive costs increase with longer extreme heat events in Collembola**

Table of contents

| Fig. S1 | Effects of extreme heat duration on the proportion of surviving adults |
| --- | --- |
| Fig. S2 | Egg production during the extreme heat phase |
| Fig. S3 | Effects of extreme heat duration on egg diameter |
| Table S1 | Output of egg production GLM |
| Table S2 | Results of the post hoc analysis of egg production GLM |
| Table S3 | Output of hatchling production GLM |
| Table S4 | Results of the post hoc analysis of hatchling production GLM |
| Table S5 | Output of clutch size GLMM |
| Table S6 | Results of the post hoc analysis of clutch size GLMM |
| Table S7 | Output of number of clutches GLM |
| Table S8 | Results of the post hoc analysis of number of clutches GLM |


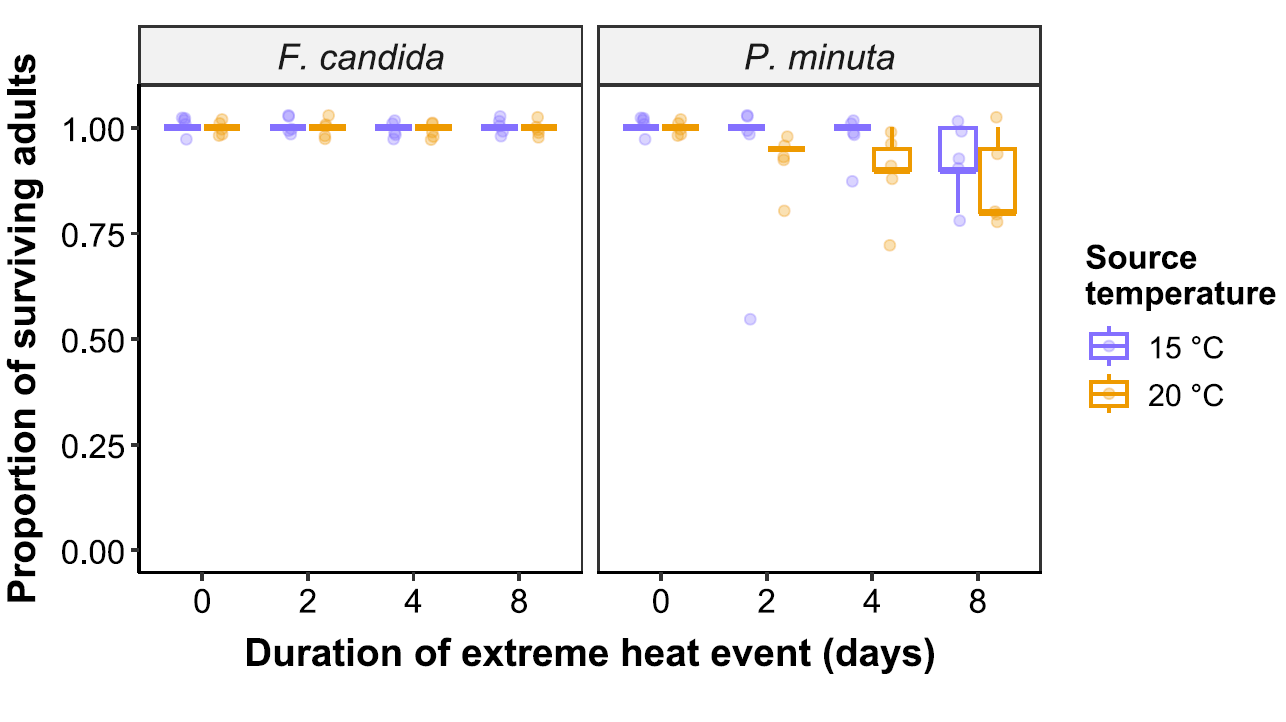


**Fig. S1.** Proportion of surviving adults of *Folsomia candida* and *Proisotoma minuta* at the end of the recovery phase. Boxplots show the median, 25^th^ and 75^th^ quantiles; and whiskers indicate 1.5 times the interquantile range. Colours designate different source temperatures at which the adults used in the experiments were raised: 15 °C (blue) or 20 °C (orange). Faded points represent raw data. In *P. minuta*, the survival of adults did not depend neither on the duration of the extreme heat events (binomial GLM slope ± SE: -0.142 ± 0.347; *P =* 0.682), the source temperature (difference in GLM intercepts between source temperatures ± SE: -0.256 ± 2.544; *P =* 0.920), or their interaction (-0.057 ± 0.448; *P =* 0.899).


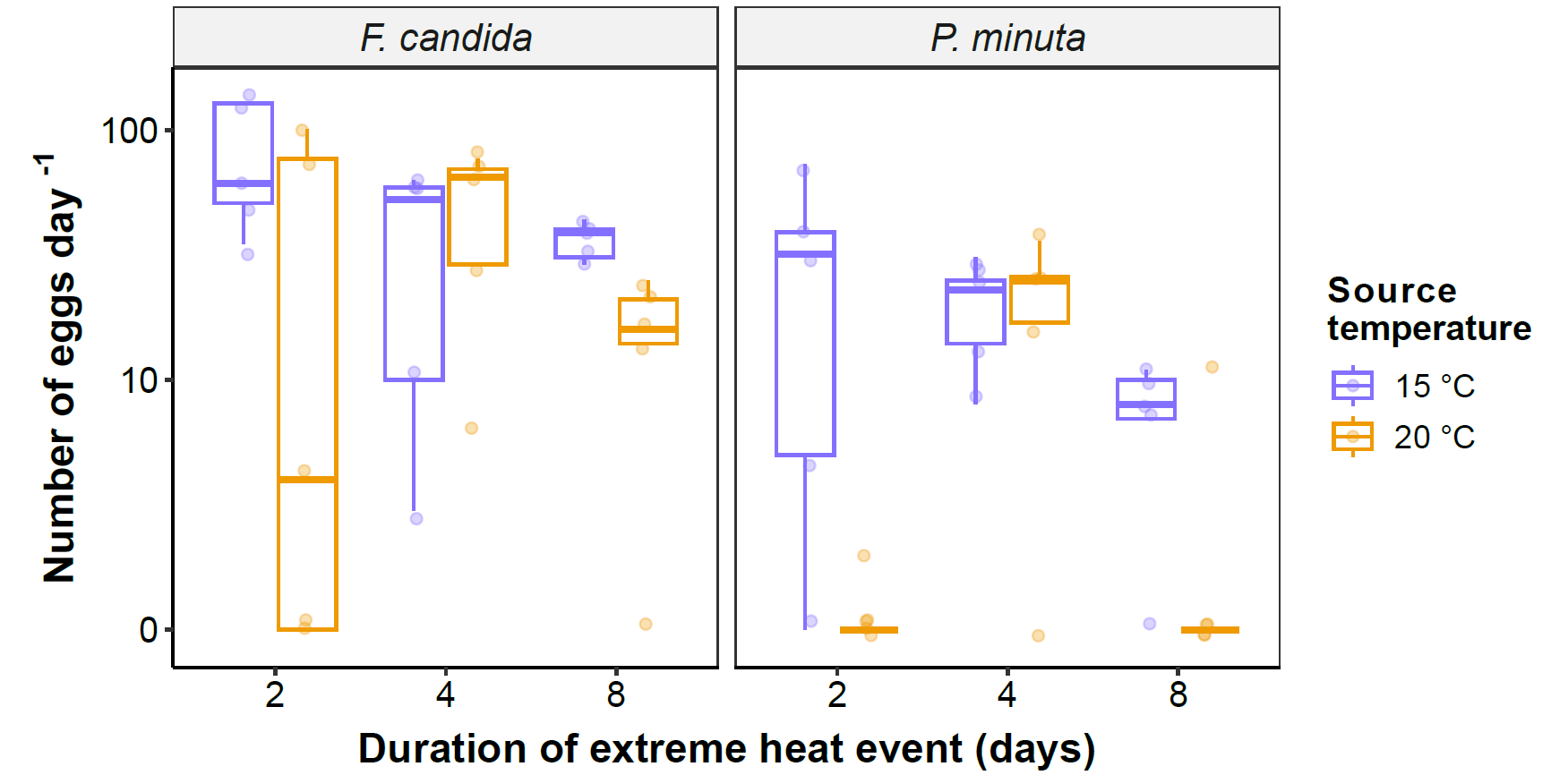


**Fig. S2.** Daily egg production during the extreme heat phase. Boxplots show the median, 25^th^ and 75^th^ quantiles; and whiskers indicate 1.5 times the interquantile range. Colours designate different source temperatures at which the adults used in the experiments were raised: 15 °C (blue) or 20 °C (orange). Faded points represent raw data. Note that only the eggs laid during the time of exposure to extreme heat conditions were counted (and later removed from the experimental units), which means that egg production in the 0-day treatment was not assessed during the extreme heat phase. This also implies that treatments with longer extreme heat events (e.g., 8-day) provided more time for multiple egg laying events compared to the shorter extreme heat events (e.g., 2-day). For this reason, a formal analysis of egg production *during* extreme heat events of varying duration is not appropriate, since the deleterious effects of the accumulation of heat stress can be confounded with the prolonged opportunities for egg laying during longer heat events.


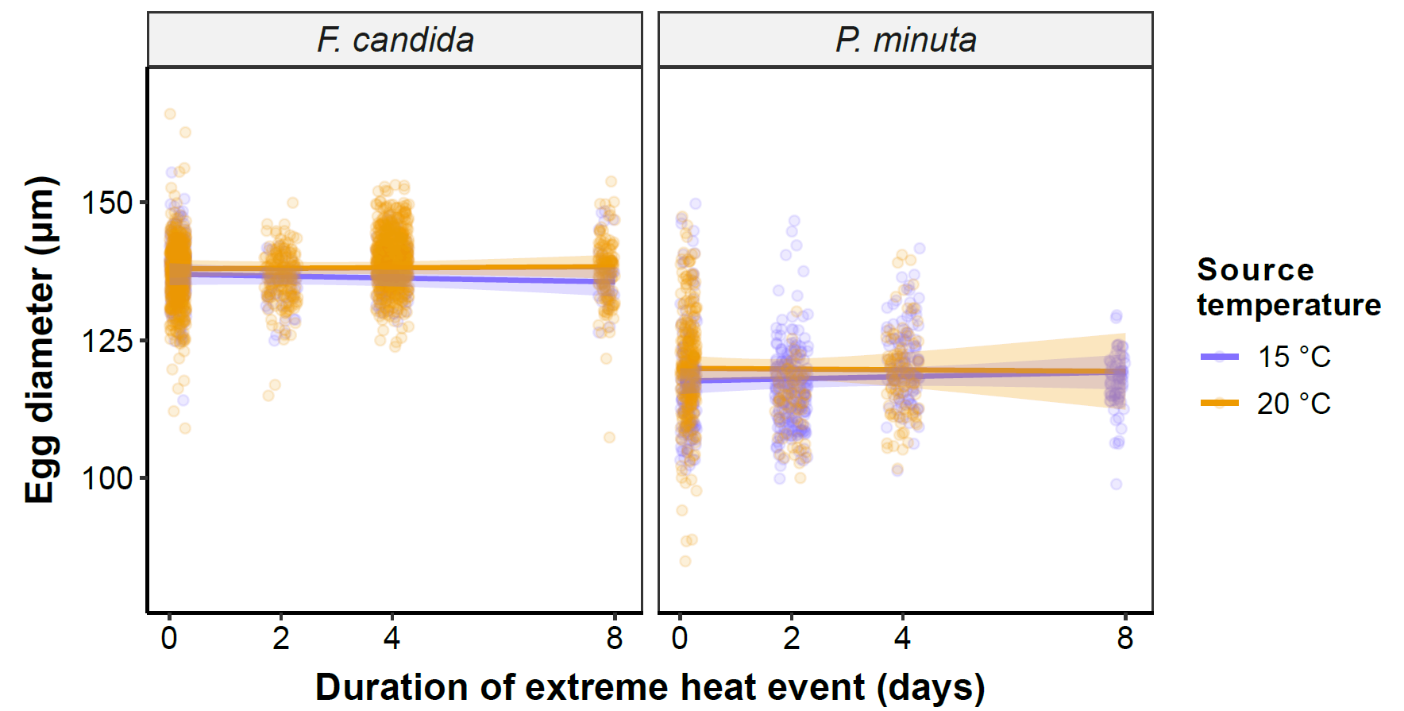


**Fig. S3.** Predicted egg diameter with 95% confidence intervals (CI) in response to extreme heat events of increasing duration. Colours designate different source temperatures at which the adults used in the experiments were raised: 15 °C (blue) or 20 °C (orange). Faded points represent raw data. The numbers of eggs measured in *Folsomia candida* were *N* = 750 (15 °C) and *N* = 2229 (20 °C), while those in *Proisotoma minuta* were *N* = 808 (15 °C) and *N* = 667 (20 °C).

**Table S1.** Output of the generalized linear model (GLM) with negative binomial distribution used to evaluate the interactive effects of the duration of extreme heat events, source temperature, and Collembola species on the number of eggs at the recovery phase. Parameter estimates with 95% confidence intervals (CI) are provided. Significant p-values (*P* < 0.05) are highlighted in bold.

| Number of eggs (log scale) | | |
| --- | --- | --- |
| Predictors | Estimate [95% CI] | *P* |
| (Intercept) | 4.10 [3.40;  4.80] | **<0.001** |
| Stress duration | -0.08 [-0.24; 0.08] | 0.301 |
| Source temp. (20 °C) | 1.14 [0.32; 1.96] | **0.006** |
| Species (*Proisotoma minuta*) | -0.40 [-1.41; 0.61] | 0.438 |
| Stress duration ×  Source temp. (20 °C) | -0.10 [-0.31; 0.10] | 0.328 |
| Stress duration ×  Species (*P. minuta*) | -0.07 [-0.31; 0.18] | 0.583 |
| Source temp. (20 °C) ×  Species (*P. minuta*) | -0.84 [-2.21; 0.53] | 0.231 |
| Stress duration × Source temp. (20 °C) × Species (*P. minuta*) | -0.22 [-0.67; 0.22] | 0.323 |
| Observations | 80 | |

**Table S2.** Results of the post hoc analysis from the GLM used to evaluate the interactive effects of the duration of extreme heat events, source temperature, and Collembola species on the number of eggs at the recovery phase. The slopes of the relationship between the number of eggs and extreme heat duration (obtained with the function *emtrends* of the R package *emmeans*; Lenth, 2024) and their 95% confidence intervals (CI) are provided. Those slopes whose confidence intervals do not overlap zero (i.e., indicating significant effects of extreme heat duration) are highlighted in bold.

| Number of eggs (log scale) | | | | | |  |
| --- | --- | --- | --- | --- | --- | --- |
| Species | Source temperature | Intercept [95% CI] | Slope extreme heat duration [95% CI] | | | |
|  |  |  |  |  |  | |
| *Folsomia candida* | 15 °C | 4.101 [3.405; 4.797] | -0.084 [-0.244; 0.075] | | | |
|  | 20 °C | 5.241 [4.778; 5.704] | **-0.186 [-0.317; -0.056]** | | | |
|  |  |  |  |  |  | |
| *Proisotoma minuta* | 15 °C | 3.701 [2.919; 4.484] | -0.153 [-0.339; 0.033] | | | |
|  | 20 °C | 4.002 [3.171; 4.834] | **-0.479** **[-0.828; -0.129]** | | | |

**Table S3.** Output of the GLM with negative binomial distribution used to evaluate the interactive effects of the duration of extreme heat events, source temperature, and Collembola species on the number of hatchlings at the hatchlings phase. Parameter estimates with 95% confidence intervals (CI) are provided. Significant p-values (*P* < 0.05) are highlighted in bold.

| Number of hatchlings (log scale) | | |
| --- | --- | --- |
| Predictors | Estimate [95% CI] | *P* |
| (Intercept) | 3.21 [2.36; 4.07] | **<0.001** |
| Stress duration | -0.53 [-0.93; -0.13] | **0.009** |
| Source temp. (20 °C) | -0.00 [-1.05; 1.05] | 0.997 |
| Species (*Proisotoma minuta*) | 0.40 [-0.64; 1.44] | 0.453 |
| Stress duration ×  Source temp. (20 °C) | 0.45 [0.02; 0.88] | **0.039** |
| Stress duration ×  Species (*P. minuta*) | 0.32 [-0.11; 0.75] | 0.143 |
| Source temp. (20 °C) ×  Species (*P. minuta*) | -0.49 [-1.95; 0.96] | 0.506 |
| Stress duration × Source temp. (20 °C) × Species (*P. minuta*) | -0.40 [-0.90; 0.10] | 0.114 |
| Observations | 80 | |

**Table S4.** Results of the post hoc analysis from the GLM used to evaluate the interactive effects of the duration of extreme heat events, source temperature, and Collembola species on the number of hatchlings at the hatchlings phase. The slopes of the relationship between the number of hatchlings and extreme heat duration (obtained with the function *emtrends* of the R package *emmeans*; Lenth, 2024) and their 95% confidence intervals (CI) are provided. Those slopes whose confidence intervals do not overlap zero (i.e., indicating significant effects of extreme heat duration) are highlighted in bold.

| Number of hatchlings (log scale) | | | | | |  |
| --- | --- | --- | --- | --- | --- | --- |
| Species | Source temperature | Intercept [95% CI] | Slope extreme heat duration [95% CI] | | | |
|  |  |  |  | | | |
| *Folsomia candida* | 15 °C | 3.211 [2.355; 4.067] | **-0.533** **[-0.931; -0.134]** | | | |
|  | 20 °C | 3.209 [2.514; 3.904] | -0.083 [-0.240; 0.074] | | | |
|  |  |  |  |  |  | |
| *Proisotoma minuta* | 15 °C | 3.609 [2.959; 4.259] | **-0.212** **[-0.378; -0.046]** | | | |
|  | 20 °C | 3.114 [2.304; 3.925] | -0.166 [-0.368; 0.037] | | | |

**Table S5.** Output of the generalized linear mixed-effects model (GLMM) with negative binomial distribution used to evaluate the interactive effects of the duration of extreme heat events, source temperature, and Collembola species on the clutch sizes (i.e., number of eggs in each clutch). Only clutches containing at least 10 eggs were considered in this analysis. Plate ID was set as a random effect in the models to account for multiple observations in the same experimental unit. Parameter estimates with 95% confidence intervals (CI) are provided, as well as marginal and conditional R^2^ (Nakagawa and Schielzeth, 2013). Significant p-values (*P* < 0.05) are highlighted in bold.

| Clutch size (log scale) | | |
| --- | --- | --- |
| Predictors | Estimate [95% CI] | *P* |
| (Intercept) | 3.37 [3.10; 3.64] | **<0.001** |
| Stress duration | -0.03 [-0.13; 0.06] | 0.486 |
| Source temp. (20 °C) | 0.11 [-0.23; 0.44] | 0.529 |
| Species (*Proisotoma minuta*) | 0.10 [-0.30; 0.49] | 0.635 |
| Stress duration ×  Source temp. (20 °C) | -0.02 [-0.13; 0.09] | 0.715 |
| Stress duration ×  Species (*P. minuta*) | -0.07 [-0.20; 0.07] | 0.345 |
| Source temp. (20 °C) ×  Species (*P. minuta*) | -0.10 [-0.63; 0.42] | 0.700 |
| Stress duration × Source temp. (20 °C) × Species (*P. minuta*) | -0.05 [-0.26; 0.16] | 0.646 |
| N _ID_ | 52 | |
| Observations | 231 | |
| Marginal R^2^ / Conditional R^2^ | 0.083 / 0.176 | |

**Table S6.** Results of the post hoc analysis from the GLMM used to evaluate the interactive effects of the duration of extreme heat events, source temperature, and Collembola species on the clutch sizes (i.e., number of eggs in each clutch). The slopes of the relationship between clutch size and extreme heat duration (obtained with the function *emtrends* of the R package *emmeans*; Lenth, 2024), their 95% confidence intervals (CI). Those slopes whose confidence intervals do not overlap zero (i.e., indicating significant effects of extreme heat duration) are highlighted in bold.

| Clutch size (log scale) | | | | |  |
| --- | --- | --- | --- | --- | --- |
| Species | Source temperature | Intercept [95% CI] | Slope extreme heat duration [95% CI] | | |
|  |  |  |  |  | |
| *Folsomia candida* | 15 °C | 3.370 [3.099; 3.642] | -0.034 [-0.129; 0.061] | | |
|  | 20 °C | 3.478 [3.276; 3.681] | -0.054 [-0.108; 0.000] | | |
|  |  |  |  |  | |
| *Proisotoma minuta* | 15 °C | 3.466 [3.178; 3.755] | **-0.099** **[-0.195; -0.003]** | | |
|  | 20 °C | 3.471 [3.195; 3.748] | **-0.168 [-0.314; -0.021]** | | |

**Table S7.** Output of the GLM with negative binomial distribution used to evaluate the interactive effects of the duration of extreme heat events, source temperature, and Collembola species on the number of clutches (i.e., proxy of the frequency of reproductive events). Only clutches containing at least 10 eggs were considered in this analysis. Parameter estimates with 95% confidence intervals (CI) are provided. Significant p-values (*P* < 0.05) are highlighted in bold.

| Number of clutches (log scale) | | |
| --- | --- | --- |
| Predictors | Estimate [95% CI] | *P* |
| (Intercept) | 0.76 [0.08; 1.45) | **0.030** |
| Stress duration | -0.47 [-0.80; -0.15) | **0.005** |
| Source temp. (20 °C) | 0.71 [-0.14; 1.57) | 0.101 |
| Species (*Proisotoma minuta*) | -0.16 [-1.17; 0.85) | 0.756 |
| Stress duration ×  Source temp. (20 °C) | 0.27 [-0.08; 0.63) | 0.133 |
| Stress duration ×  Species (*P. minuta*) | 0.16 [-0.26; 0.58) | 0.456 |
| Source temp. (20 °C) ×  Species (*P. minuta*) | -0.61 [-1.95; 0.73) | 0.371 |
| Stress duration × Source temp. (20 °C) × Species (*P. minuta*) | -0.43 [-0.98; 0.13) | 0.134 |
| Observations | 80 | |

**Table S8.** Results of the post hoc analysis from the GLM used to evaluate the interactive effects of the duration of extreme heat events, source temperature, and Collembola species on the number of clutches (i.e., proxy of the frequency of reproductive events). The slopes of the relationship between the number of clutches and extreme heat duration (obtained with the function *emtrends* of the R package *emmeans*; Lenth, 2024) and their 95% confidence intervals (CI) are provided. Those slopes whose confidence intervals do not overlap zero (i.e., indicating significant effects of extreme heat duration) are highlighted in bold.

| Number of clutches (log scale) | | | | | |  |
| --- | --- | --- | --- | --- | --- | --- |
| Species | Source temperature | Intercept [95% CI] | Slope extreme heat duration  [95% CI] | | | |
|  |  |  |  |  |  | |
| *Folsomia candida* | 15 °C | 0.760 [0.075; 1.446] | **-0.471** **[-0.797; -0.146]** | | | |
|  | 20 °C | 1.475 [0.966; 1.984] | **-0.200 [-0.340; -0.059]** | | | |
|  |  |  |  |  |  | |
| *Proisotoma minuta* | 15 °C | 0.600 [-0.144; 1.343] | **-0.310** **[-0.578; -0.043]** | | | |
|  | 20 °C | 0.704 [-0.004; 1.411] | **-0.464** **[-0.796; -0.132]** | | | |
